# A suitable (wide-range + linear) temperature sensor based on Tm^3+^ ions

**DOI:** 10.1038/s41598-017-14535-1

**Published:** 2017-10-26

**Authors:** A. R. Zanatta, D. Scoca, F. Alvarez

**Affiliations:** 1 Instituto de Física de São Carlos, USP, São Carlos, 13560-970 SP Brazil; 2 Instituto de Física Gleb Wataghin, UNICAMP, Campinas, 13083-859 SP Brazil

## Abstract

Future advances in the broad fields of photonics, (nano-)electronics or even theranostics rely, in part, on the precise determination and control, with high sensitivity and speed, of the temperature of very well-defined spatial regions. Ideally, these temperature-sensors (T-sensors) should produce minimum (or no) disturbance in the probed regions, as well as to exhibit good resolution and significant dynamic range. Most of these features are consistent with the sharp and distinctive optical transitions of trivalent rare-earth (RE^3+^) ions that, additionally, are susceptible to their local environment and conditions. Altogether, these aspects form the basis of the present work, in which we propose a new T-sensor involving the light emission of trivalent thulium ions (Tm^3+^) embedded into crystalline TiO_2_. The optical characterization of the TiO_2_:Tm^3+^ system indicated a Tm^3+^-related emission at ~676 nm whose main spectral features are: (1) a temperature-induced wavelength shift of −2.2 pm K^−1^, (2) a rather small line-width increase over the ~85–750 K range, and (3) minimum data deconvolution-processing. The study also included the experimental data of the well-established pressure- and T-sensor ruby (Al_2_O_3_:Cr^3+^) and a comprehensive discussion concerning the identification and the excitation-recombination mechanisms of the Tm^3+^-related transitions.

## Introduction

Along with its fairly intuitive (though not simple) concept, the physical quantity temperature is present in most of the processes taking place in real world – with exceptional examples ranging from the atomic to the outer-space environments^1,2^. Indeed, temperature is essential in many biological-chemical-physical processes influencing their activation-maintenance-interruption and, most importantly, determining their efficiency and final products^3^. Notwithstanding its omnipresence and importance, there is no universal temperature sensor (T-sensor) that comply with the specificities of all (or most of) the probed objects in terms of: range, sensitivity, precision, time response, spatial resolution, and degree of interference, for example.

T-sensors can be separated into primary or secondary systems as they provide absolute temperature values (following state equations, for example) or rely on previous calibration, respectively. Both of them derive from T-induced variations of specific materials properties such as those involving: physical aspects (dimensions, pressure, velocity, density), electrical characteristics (resistance, difference of potential, Seebeck or Peltier effects), optical radiation (light absorption-emission-polarization, index of refraction, blackbody emission), and magnetic features (chemical shift, susceptibility)^4^. Within these, modern applications require T-sensors able to provide non-contact temperature measurements of regions down to the micrometer range^5,6^, as well as reliability, high sensitivity, speed, and linearity over a considerable temperature range^7^. Most of the above aspects are fulfilled by the optically-based methods, in which the temperature can be achieved by means of spectral analysis (wavelength position, intensity, shape, width, lifetime, or polarization dependence) of certain optical transitions.

A classical example of such optically-based T-sensor is ruby (Al_2_O_3_ doped with Cr^3+^ ions) that is a standard pressure-sensor as well^8,9^. For that purpose, the light emission at ~693 nm (corresponding to the R_1_ transition of Cr^3+^ ions) is frequently used^10^, rendering quite impressive wavelength shifts, *i*.*e*.: ${\rm{\Delta }}{\lambda }_{{{\rm{R}}}_{1}}({\rm{T}})$ = +7.7 pm K^−1^ ^8^, and ${\rm{\Delta }}{\lambda }_{{{\rm{R}}}_{1}}({\rm{P}})$ = +36.5 pm kbar^−1^ ^11^. Even though these outstanding figures and widespread use, as far as temperature measurements are concerned, the spectral features provided by ruby crystals lack from good linearity and spatial resolution. More specifically, R_1_ ruby-related wavelength shift is linear only in the ~300–600 K range^8^, and no successful temperature-mapping (or -imaging) experiments were reported to date.

In addition to ruby, other optically-based T-sensors include the use of organic dyes^12,13^, semiconductor quantum-dots^14,15^, rare-earth ions^16–19^, and luminescent (bio-)polymers^20,21^, for example. As expected, each of the above T-sensors are best suited for a specific range of temperatures and their sensitivities depend on the optical spectrum characteristics (Table 2 of ref.^5^, for example, presents an excellent overview with the typical sensitivities and range of temperatures of most of the above T-sensors).

Within this context, the T-sensors based on rare-earth ions are of special importance because of features such as: constant thermal sensitivity (as denoted by a linear behavior over a considerable range of temperatures), and no photo-degradation nor stability problems (in contrast to organic-based materials and quantum-dot structures). Furthermore, the list of rare-earth ions allows different options of photon excitation-emission (practically along the entire UV-VIS-NIR range), and compatibility with various classes of (in)organic materials (in the form of solids, liquids, nano- or micro-composites etc.) that, traditionally, have been considered for the development of rare-earth-based light-emitting sources. This is particularly true for trivalent thulium (Tm^3+^) ions that, so far, had presented laser action at multiple wavelengths^22,23^, as well as a few examples of their potential as a T-sensor^24^. In this last case, however, the T-sensor was based on the lifetime decay of the Tm^3+^-related integrated signal in the 1000–1700 nm NIR region, after excitation with 800 nm photons. According to the experimental results, excellent fluorescence stability and good temperature sensitivity (~3 μs K^−1^), in the ~298–1675 K range, were achieved from a Tm^3+^-doped YAG (or Y_3_Al_5_O_12_) optical fiber^25,26^.

Despite the use of Tm^3+^ ions, we adopted a completely different approach in the present contribution. More specifically: the optically-active Tm^3+^ ions were embedded into a crystalline TiO_2_ film, and the desired temperature was determined from the shift experienced by the Tm^3+^-related emission at ~676 nm. Moreover: such wavelength shift proved to be rather linear (−2.2 pm K^−1^) in the ~85–750 K range, and required almost no spectra deconvolution-analysis. Because this is the first time the wavelength shift of the Tm^3+^-related is being proposed as a T-sensor, the work also contains a comprehensible discussion involving the main optical excitation-recombination mechanisms of the Tm^3+^ ions. A few practical applications that could be benefited from the present Tm^3+^-based T-sensor are outlined as well.

## Results and Discussion

**Figure 1:**
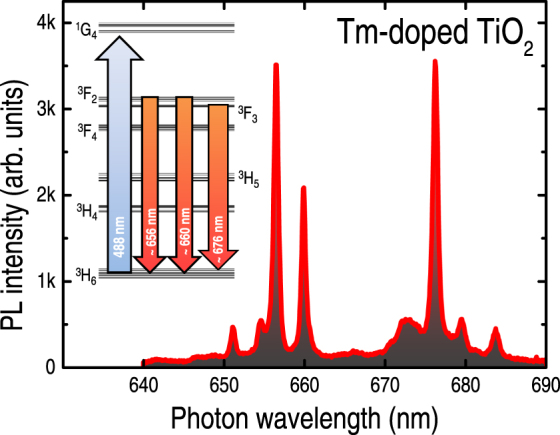
Photoluminescence (PL) spectra of a Tm-doped crystalline TiO_2_ sample. The spectrum was achieved at room-temperature (298 K) by exciting the sample with 488.0 nm photons (~2 mW μm^−2^ power density). The inset contains a simplified energy levels diagram of the Tm^3+^ ion illustrating its optical excitation (488.0 nm) and transitions at ~656, 660, and 676 nm.

The photoluminescence (PL) spectrum of the TiO_2_:Tm^3+^ sample is displayed in Fig. 1 The spectrum was obtained at room-temperature by exciting the sample with 488.0 nm photons, and shows three Tm^3+^-related optical transitions in the 640–690 nm wavelength range, along with a Tm^3+^ energy levels diagram (inset of Fig. 1).Considering the absence of spectroscopic data of Tm^3+^ ions in the TiO_2_ matrix, the present PL assignment was based on previous works involving Tm^3+^ embedded into YCl_3_^27^ and KYb(WO_4_)_2_ crystals^28^. Furthermore, for simplicity reasons, only three Stark levels have been assumed, which were associated to the lowest (index 0), central (index 1), and highest (index 2) energy values found in the studies of YCl_3_:Tm^3+^ and KYb(WO_4_)_2_:Tm^3+^. According to this procedure, the main PL lines observed in Fig. 1 are due to the following transitions: ^3^F_2,2_ → ^3^H_6,2_ (at ~656 nm), ^3^F_2,0-1_ → ^3^H_6,2_ (at ~660 nm), and ^3^F_3,0-1_ → ^3^H_6,0-1_ (at ~676 nm) [Supplementary Figure S1]. Likewise, the photon excitation with 488.0 nm photons is quasi-resonant with the ^3^H_6,0-2_ → ^1^G_4,0-2_ transition.

In addition to these, other optical emissions were observed in the 500–850 nm interval: either due to Tm^3+^ ions (at ~560 and 790 nm, ascribed to the ^3^F_3_ → ^3^H_5_, and ^1^G_4_ → ^3^H_5_ or ^3^F_4_ → ^3^H_6_ transitions, respectively), or originating from the TiO_2_ matrix (>800 nm)^29^.

**Figure 2:**
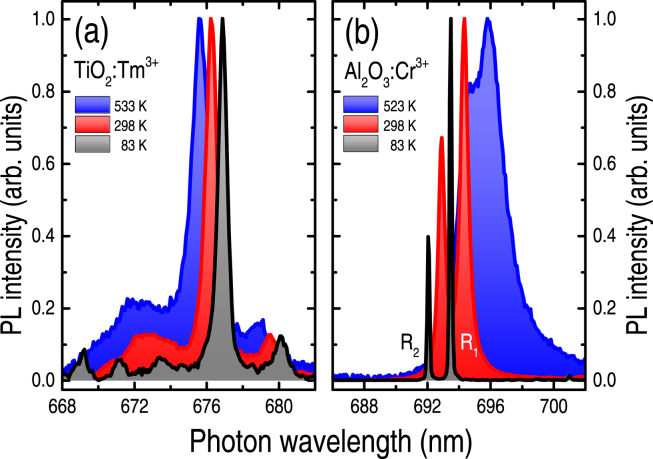
Photoluminescence spectra (taken at the temperatures indicated in the figure) of: (**a**) a Tm-doped TiO_2_ sample, and (**b**) natural ruby (Al_2_O_3_:Cr^3+^) crystal. All measurements were performed after keeping the samples at the desired temperature for ~2 min. The spectra were normalized for comparison purposes. Notice the spectral changes (wavelength and line-width) experienced by the Tm^3+^- and Cr^3+^-related transitions as induced by the temperature of measurement.

As indicated by the room-temperature spectrum of Fig. 1, the Tm^3+^-related optical emissions are well-defined, background-free, and rather sharp. Nonetheless, the most remarkable feature of the Tm^3+^-related signal involves the temperature-induced wavelength shift of the ^3^F_3,0-1_ → ^3^H_6,0-1_ (at ~676 nm) transition, without major changes in the overall spectrum (Fig. 2). Such behavior is in contrast with that exhibited by the traditional optically-based T-sensor ruby^8^, for example, in which case the spectral line-width and, consequently, temperature precision are highly affected at increasing temperatures.

The analysis of the Tm^3+^-related PL(T) spectra indicate that the ^3^F_3,0-1_ → ^3^H_6,0-1_ transition experienced a linear, wavelength shift dλ/dT of −2.2 pm K^−1^ (or +0.048 cm^−1^ K^−1^), with a 1.25 nm (or 55 cm^−1^) line-width increase, along the entire ~85–750 K measured range. Also, the PL signal intensity I_PL_ changed for only two orders of magnitude and the spectral analysis required almost no data processing, except for the fitting of a Lorentzian function to the ^3^F_3,0-1_ → ^3^H_6,0-1_ transition signal [Supplementary Figure S2].

Analogously, for the R_1_ and R_2_ ruby lines (measured and analyzed in the very same way): (1) the wavelength shifts were higher (+7.7 pm K^−1^ or −0.16 cm^−1^ K^−1^), but presented a linear behavior only in the ~300–600 K interval, (2) from ~85 to 750 K, the corresponding line-widths increase were R_1_ ~ 6.8 nm (or 280 cm^−1^) and R_2_ ~ 2.6 nm (or 109 cm^−1^), (3) the I_PL_ signal experienced a three orders of magnitude variation, and (4) above ~400 K there was a considerable overlapping of the R_1_-R_2_ lines which influenced the wavelength accuracy [Supplementary Figure S3].

**Figure 3:**
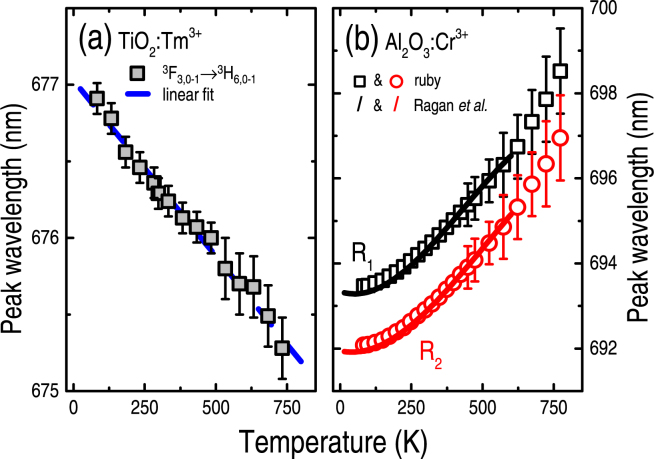
Peak wavelength as a function of temperature (~85–750 K) due to: (**a**) Tm^3+^ ions embedded into crystalline (Anatase) TiO_2_ films (^3^F_3,0-1_ → ^3^H_6,0-1_ transition), and (**b**) Cr^3+^ ions embedded into ruby or Cr-doped Al_2_O_3_ crystal (R_1_ and R_2_ lines corresponding to the ^2^E → ^4^A_2_ transition). All points refer to experimental data obtained in the present work. The dashed blue (solid black and red) lines account for the mathematical fitting of the experimental data (the expression given by Ragan *et al*.^8^). The error bars comprise uncertainties due to experimental acquisition and data analysis.

**Figure 4:**
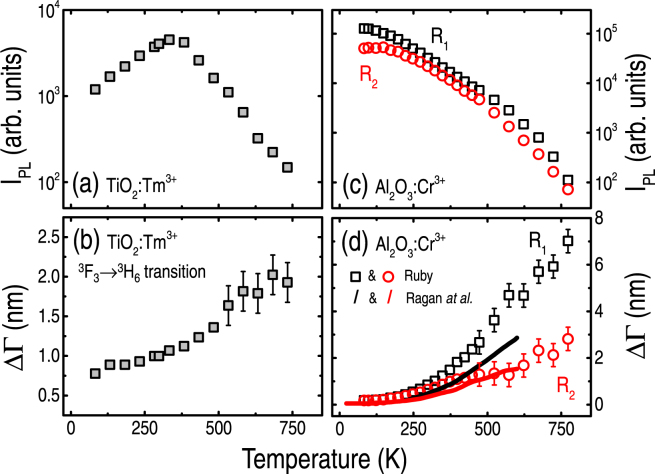
Photoluminescence intensity (I_PL_) and transition line-width (ΔΓ), in the ~85–750 K temperature range, due to: (**a**,**b**) Tm^3+^ ions (^3^F_3,0-1_ → ^3^H_6,0-1_ transition), and (**c**,**d**) Cr^3+^ ions (R_1_ and R_2_ lines corresponding to the ^2^E → ^4^A_2_ transition). All points refer to data obtained in the present work, following 488.0 nm excitation (~2 mW μm^−2^ power density). The black and red solid lines refer to the expression given by Ragan *et al*.^8^. Error bars took into account uncertainties involving spectra acquisition and data analysis.

Most of the above features are displayed in Figs 3 and 4, in which the error bars took into account: the experimental resolution (~1 cm^−1^), different measurement runs, and data processing-analysis (involving changes due to background removal, for example).

Figure 3 shows the peak wavelength of the Tm^3+^ and Cr^3+^ ions, as experimentally determined in the ~85–750 K range. The figure also presents the mathematical fittings of the Tm^3+^-related experimental data [${\lambda }_{{{\rm{Tm}}}^{3+}}({\rm{T}})$ = 676.9 − 2.2 × 10^−3^ T nm], as well as those obtained by Ragan *et al*.^8^ in ruby [${\lambda }_{{{\rm{R}}}_{1}}({\rm{T}})$ = 693.34 − 2.17 × 10^−3^ T + 2.31 × 10^−5^ T^2^ − 1.78 × 10^−7^ T^3^ nm and ${\lambda }_{{{\rm{R}}}_{2}}({\rm{T}})$ = 691.94 − 1.44 × 10^−3^ T + 1.86 × 10^−5^ T^2^ − 1.21 × 10^−8^ T^3^ nm – in the 0–300 K interval]. Not only the gradient and linearity of the curves are different but, interestingly, the wavelength shifts presented by both ions are contrary: negative for Tm^3+^ ions (blue-shift), and positive for Cr^3+^ (red-shift).

All of these characteristics derive from the properties of each ion as well as from the ion-host interaction and, part of them, were reported in the literature^30–32^. Basically, when embedded into a crystal host, impurities (like the Tm^3+^ or Cr^3+^ ions, for example) move around their equilibrium positions giving rise to lattice vibrations. Optical transitions between any two levels of these impurities can be brought about in, at least, two ways: (a) a direct or electron-phonon EP process (transition from one level to another such that any energy difference is compensated by phonon absorption or emission, to allow the transition), and (b) a Raman process (the transition is made possible by energy variations due to phonon scattering).

As expected, both EP and Raman processes are dependent on temperature and, ultimately, affects the wavelength and line-width of the transitions. In the former case, the T-induced wavelength shifts are associated with variations of the crystal field energy because of local lattice vibrations. In the latter, the line-width changes as a result of the transitions between the crystal field levels stimulated by interaction with the acoustical phonon field. Roughly, the T-induced variations in the spectral characteristics of any transition can be described by^31^:

1 $${\rm{E}}({\rm{T}})={\rm{E}}(0)+{{\rm{E}}}_{{\rm{direct}}}({\rm{T}})+{{\rm{E}}}_{{\rm{Raman}}}({\rm{T}})\,{\rm{and}}\,{\rm{\Gamma }}({\rm{T}})={\rm{\Gamma }}(0)+{{\rm{\Gamma }}}_{{\rm{direct}}}({\rm{T}})+{{\rm{\Gamma }}}_{{\rm{Raman}}}({\rm{T}})$$

where E and Γ stand for the energy and line-width of the transition, respectively. Moreover: the first terms [E(0) and Γ(0)] account for the T-independent or static-like interactions (crystal field interaction, nephelauxetic effect, strain-stress, for example), the index “direct” stand for the direct processes (EP interactions and those ones associated with non-harmonic effects like thermal expansion-compression^33^), and the index “Raman” denotes the processes involving the scattering of phonons. The formal description of the E(T) and Γ(T) quantities are based on the Hamiltonian of the impurity-phonon system and the Debye model for the phonon distribution. It involves a lot of adjustable-unknown parameters and, in most of the cases, it reproduces the experimental results at the expense of unreliable-conflicting values (Debye temperature and phonon frequency different from those available in literature, different coupling factors for the very same transition, different E(0) and Γ(0) values for the very same material etc.).

In spite of this limitation, the model is very instructive to indicate the origin of the observed red- and blue-shifts. According to the model, the red-shift is typical of impurity–matrix systems in which the EP interactions and/or phonon scattering processes are predominant, as verified in ruby (Al_2_O_3_:Cr^3+^)^30^, and YAG:Cr^3+^ ^34^. Blue-shifts, on the contrary, are typical of the rare-earth ions because of the strong non-harmonic influence^31,33,35,36^. In fact, the primacy of the EP interactions and/or phonon-scattering processes are consistent with the great changes observed in the I_PL_ and line-width of the Cr^3+^-related transitions [Fig. 4(c,d)], that are also susceptible to strain-stress and concentration details^37^.

Concerning the peculiar I_PL_ behavior presented by the TiO_2_:Tm^3+^ sample [Fig. 4(a)], it originates because of variations in the energy of the Tm^3+^ levels and the quasi-resonant photon excitation process. More precisely, I_PL_ is highest when photon absorption is maximum, *i*.*e*., around room-temperature due to the almost perfect match between the ^3^H_6,0-2_ → ^1^G_4,0-2_ transition and the 488.0 nm photon energy. Below, or above, room-temperature the energy of the ^1^G_4,0-2_ levels change such that the photon absorption process is incomplete, rendering some decrease of I_PL_. Likewise, the T-induced energy variation of certain levels is the origin of the wavelength shift values presented by the Tm^3+^ and Cr^3+^ ions.

Despite their inherent energy levels arrangement, both Tm^3+^ and Cr^3+^ ions are influenced by their local atomic surroundings which, in essence, are decided by external agents like temperature, pressure, electromagnetic fields etc. Such ion–host correlation is usually expressed in terms of the crystalline-field theory, that has been extensively considered in the study of rare-earth (RE) and transition-metal (TM) ions^38,39^.

According to this approach, RE^3+/2+^ ions appear to be the less affected by the so-called crystalline-field effects. It happens because of the outer 5 s and 5p orbitals that partially shield the intra-4f transitions of the RE^3+^ or RE^2+^ ions – in contrast to the TM^3+^-related transitions that take place at the vulnerable 3d orbitals. Nonetheless, the local atomic environment changes the RE^3+/2+^-related transitions, and its final effects can be enhanced depending on the ion–host characteristics, as verified in this work.

**Figure 5:**
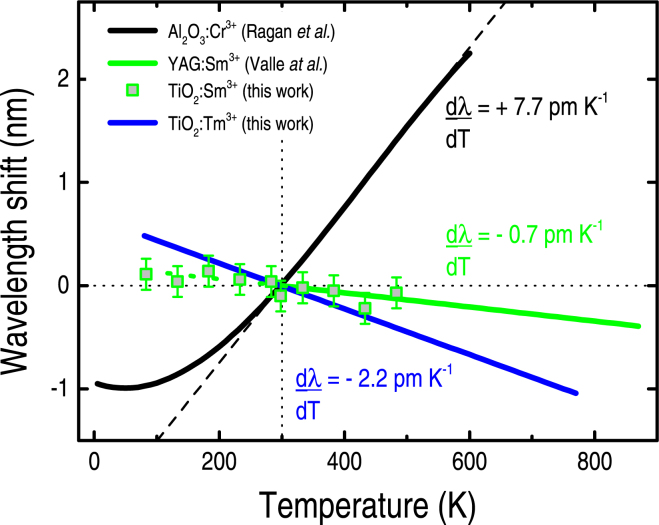
Wavelength shift (as referred to that verified at room-temperature) as a function of temperature due to: Cr^3+^ in ruby (experimental data of Ragan *et al*.^8^), Sm^3+^ when inserted in YAG crystals (Valle *et al*.^36^), and Sm^3+^ and Tm^3+^ ions embedded into crystalline Anatase TiO_2_ films (this work). The solid lines refer to the best mathematical description of the experimental data. Along with the higher dλ/dT exhibited by ruby, notice its modest range (~300–600 K) of linear variation.

Based on the present experimental results, the wavelength-shift experienced by Tm^3+^, at ~676 nm, corresponds to almost 30% that of Cr^3+^. The wavelength-shifts of the other Tm^3+^-related transitions (at ~656 and 660 nm) are well-defined and linear as well, but presented a smaller rate: dλ/dT (^3^F_2,2_ → ^3^H_6,2_) = dλ/dT (^3^F_2,0-1_ → ^3^H_6,2_) = −1.1 pm K^−1^ [Supplementary Figure S4]. These figures not only are consistent with the crystalline-field theory (in the sense that RE^3+/2+^ ions are less sensitive than TM^3+^ ions), but are similar to those achieved from other RE^3+/2+^ ions: Eu^3+^ in YAG with dλ/dt = −0.5 pm K^−1^ (or +0.015 cm^−1^ K^−1^) and a linear 293–1073 K range^40^; Sm^3+^ in YAG with dλ/dt = −0.7 pm K^−1^ (or +0.018 cm^−1^ K^−1^) and a linear 300–873 K range^36^; Sm^3+^ in TiO_2_ with dλ/dt = −0.7 pm K^−1^ (or +0.017 cm^−1^ K^−1^) and a linear 83–750 K range^41^ [Supplementary Figure S5]; Sm^2+^ in SrB_4_O_7_ with dλ/dt = −0.1 pm K^−1^ (or +0.002 cm^−1^ K^−1^) and a linear 293–673 K range^42^. The only exception to these values applies to the Tm^3+^-related transition at ~676 nm, in which dλ/dT = −2.2 pm K^−1^ (or +0.048 cm^−1^ K^−1^) is, at least, 2–3 times higher than other RE^3+/2+^ ions. This can be seen in Fig. 5 that shows the wavelength shifts of Tm^3+^ (at ~676 nm), Sm^3+^ (one of those exhibiting the highest slopes amongst the RE ions, at ~612 nm), and Cr^3+^ ions (representing the standard optically-based T-sensor).

Within the possible reasons that explain the wavelength-shift presented by the TiO_2_:Tm^3+^ sample we can mention: (a) the arrangement of Tm^3+^-related energy levels, as imposed by the TiO_2_ host; and (b) the TiO_2_ host in the form of a thin film, aggregating to the already existing T-induced crystalline-field and non-harmonic effects, those ones due to the T-induced film-substrate interaction. Though reasonable, the film-substrate interaction is a hypothesis that requires the systematic investigation of a bulk TiO_2_:Tm^3+^ sample for validation. Therefore, the main spectroscopic features of the TiO_2_:Tm^3+^ system can be summarized as: (1) TiO_2_ is a wide bandgap material that is able to produce considerable field distortion around the Tm^3+^ ions; (2) the ^3^F_2,2_ → ^3^H_6,2_, ^3^F_2,0-1_ → ^3^H_6,2_, and ^3^F_3,0-1_ → ^3^H_6,0-1_ transitions take place at ~656, 660, and 676 nm, respectively; (3) temperature determines the crystalline-field and gives rise to non-harmonic effects like thermal compression-expansion; (4) as the temperature advances, the mutual play of these effects provokes the separation of the Tm^3+^-related energy levels and, ultimately, the blue-shift of all transitions; and (5) optical transitions ending at the ^3^H_6,2_ state (*i*.*e*., ^3^F_2,2_ → ^3^H_6,2_ and ^3^F_2,0-1_ → ^3^H_6,2_) present a smaller blue-shift than those involving the lowest ^3^H_6,0-1_ ground states (^3^F_3,0-1_ → ^3^H_6,0-1_) – in accord with quantum mechanics principles^31^.

In summary, the TiO_2_:Tm^3+^ system represents a very convenient option to the ever-increasing area of optically-based temperature sensors. Along with its many interesting characteristics (linear blue-shift over a quite large T-range, visible light emission with no great line-width variation, and no need of hard spectrum processing), the system comprises the inert-biocompatible TiO_2_ matrix. Indeed, the whole procedure (sample production + optical excitation/detection + spectrum analysis) involves rather simple instrumentation which, allied to its sensitivity, suggests the potential of the TiO_2_:Tm^3+^ system as an suitable T-sensor in photonics, (nano-)electronics, theranostics^43–45^, thermal imaging-mapping etc. Moreover, in the form of a thin film, the TiO_2_:Tm^3+^ system allows its attachment (instant or permanent) to almost any solid surface – ideally in the 1–10^−9^ m range – without generating great temperature variations.

### Concluding remarks

We reported on the use of Tm^3+^ ions as an optically-based temperature-sensor. The Tm^3+^ ions were embedded into a crystalline TiO_2_ film, and the actual temperature was determined from the shift experienced by the Tm^3+^-related emission at ~676 nm. Were applicable, the work compared the spectroscopic data of the Tm^3+^ ions with those of the well-established pressure- and T-sensor ruby (Al_2_O_3_:Cr^3+^). In contrast to ruby, the experimental results indicated that the Tm^3+^-related wavelength shift is rather linear and required almost no spectra deconvolution-analysis. Also, whereas the Cr^3+^ ions showed a red-shift of +7.7 pm K^−1^, the Tm^3+^ ions exhibited a smaller (−2.2 pm K^−1^) blue-shift, though over a considerable dynamic range (83–750 K).

We also presented a comprehensive discussion concerning the Tm^3+^-related transitions as well as the T-induced factors that most affect their wavelength shift.

The present experimental results suggest the suitability of the TiO_2_:Tm^3+^ system as an optically-based T-sensor in terms of its simplicity, sensitivity, and wide linear dynamic range. Future work, however, should consider its spatial resolution as well as its real operation in photonics, (nano-)electronics, and biological applications, for example.

## Methods

The present thulium-doped TiO_2_ sample was deposited onto a crystalline silicon substrate by sputtering a high purity Titanium + Thulium solid target (properly adjusted according to the Ti and Tm relative areas and sputtering yields^46^) to generate a Tm concentration around 0.5 at.%^18^. During deposition, the Ti + Tm target was bombarded by a beam of Ar^+^ ions (1.5 keV and nominal current ~13 mA/cm^2^) that was generated by a Kaufman cell. The whole procedure was carried out in a high vacuum chamber under an atmosphere of pure oxygen (5 × 10^−4^ mbar), by keeping the crystalline silicon substrate at 473 K. Under these conditions, after 120 min, a 300 nm thick uniform film was obtained.

Subsequently, the Tm-doped TiO_2_ sample was cut into 1 cm^2^ pieces and one of them was annealed at 873 K under a flow of oxygen gas at atmospheric pressure, during 30 min. All samples – as-deposited and after thermal annealing – were investigated by X-ray photoelectron spectroscopy (XPS), X-ray diffraction (XRD), Raman spectroscopy, and photoluminescence (PL) experiments. Whereas the XPS analysis confirmed the presence of ~0.5 at.% of Tm in the TiO_2_ films, both XRD and Raman measurements indicated their amorphous to crystalline transformation (predominantly in the Anatase phase) after thermal annealing at 873 K [Supplementary Figure S6].

The PL experiments were carried out in a commercial micro-Raman setup (Renishaw RM2000) by exciting the Tm-doped TiO_2_ sample with 488.0 nm photons. The temperature-dependent spectra were obtained in the 83–773 K range by means of a computer-controlled T-stage (Linkam THMS 600). Further information concerning the sample deposition conditions (and method^47^) and characterization details can be found elsewhere^41^.

For comparison purposes, a Sm-doped TiO_2_ film (deposited and annealed following exactly the same procedure above described) and a natural ruby sample (Cr-doped Al_2_O_3_) had their photoluminescence investigated in detail. Given the high temperature sensitivity of the samples under investigation, the PL measurements were conducted at the lowest laser excitation power density (~2 mW μm^−2^). Following this condition, all observed spectral variations (wavelength shift, line-width, and signal intensity) are exempt from experimental artifacts. Accordingly, all PL(T) measurements shared the same experimental accuracy, *i*.*e*.: ~1 cm^−1^ (spectral) and <0.2 K (reproducibly achieved after 2 min of thermal stabilization).

## Electronic supplementary material

Supplementary information
