# Supplementary material for: A suitable (wide-range + linear) temperature sensor based on Tm3+ ions
Source: Sci Rep. 2017 Oct 26;7:14113. doi: 10.1038/s41598-017-14535-1 (PMC5658393; doi:10.1038/s41598-017-14535-1)
Supplement: Supplementary file 1 — Supplementary information [file 41598_2017_14535_MOESM1_ESM.pdf]

Supplementary Figures

A suitable (wide-range + linear) temperature sensor based on Tm^{3+} ions

(Zanatta, Scoca, and Alvarez)

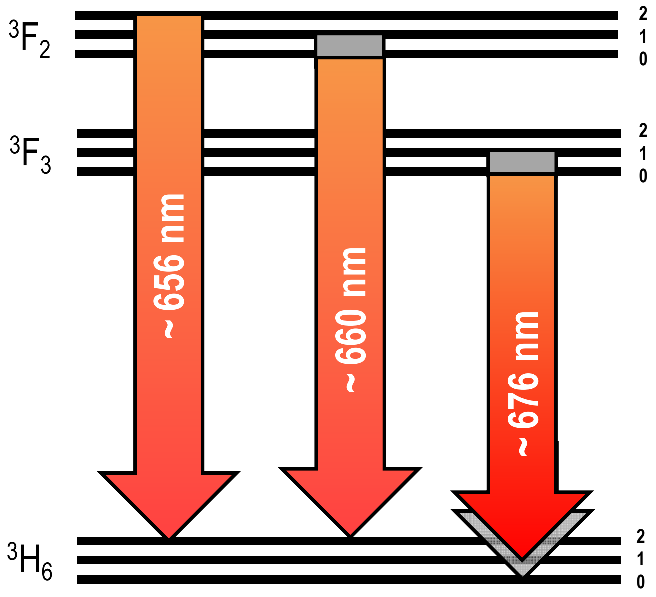

Supplementary Figure S1– Reduced energy levels diagram of Tm^{3+} ions, highlighting the $^3F_2$, $^3F_3$, and $^3H_6$ levels along with their Stark splittings: lowest (index 0), central (index 1), and highest (index 2). The optical transitions at ~ 656, 660, and 676 nm were based on the spectroscopic data of YCl_3 [Dieke, G. H. *Spectra and energy levels of rare-earth ions in crystals* (Wiley Interscience, New York NY, 1968), Chapter 13.] and $KYb(WO_4)_2$ crystals [Pujol, M. C., Guell, F., Mateos, X., Gavaldà, J., Solè, R., Massons, J., Aguiló, M., Díaz, F., Boulon, G. & Brenier, A. *Crystal growth and spectroscopic characterization of* Tm^{3+}*-doped* $KYb(WO_4)_2$ *single crystals*, *Phys. Rev. B* **66**, 144304-1-8 (2002).] doped with Tm^{3+} ions. Accordingly, the most probable emissions taking place in the $TiO_2:Tm^{3+}$ samples are, as denoted in the figure: $^3F_{2,2} \rightarrow {}^3H_{6,2}$ (at ~ 656 nm), $^3F_{2,0\text{-}1} \rightarrow {}^3H_{6,2}$ (at ~ 660 nm), and $^3F_{3,0\text{-}1} \rightarrow {}^3H_{6,0\text{-}1}$ (at ~ 676 nm).

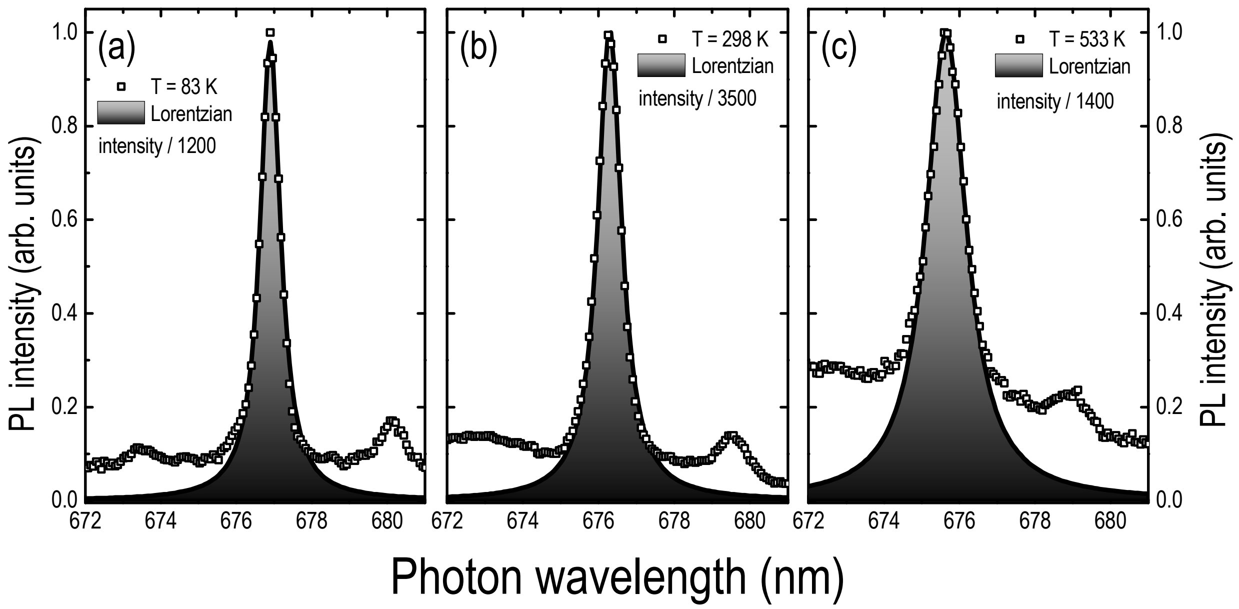

Supplementary Figure S2– Spectra fitting (with a single Lorentzian function) of the $^3F_{3,0\text{-}1} \rightarrow {}^3H_{6,0\text{-}1}$ transition due to Tm^{3+} ions in crystalline TiO_2. The fittings were taken without background removal (see Note)*.

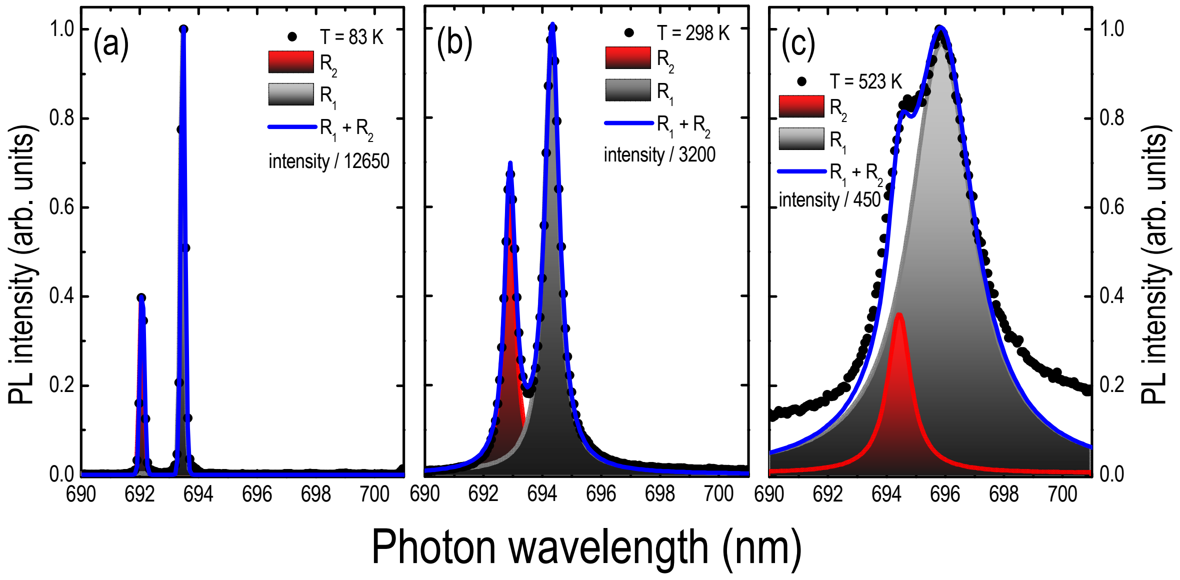

Supplementary Figure S3– Spectra fitting (with a couple of Lorentzian functions) of the Cr^{3+}-related optical emissions (R_1 and R_2 lines corresponding to the $^2E \rightarrow {}^4A_2$ transition), as obtained from natural ruby. The fittings were taken without background removal (see Note)*.

*Note– The final spectroscopic data (peak wavelength, line-width, and intensity), and respective error bars, as shown in the manuscript, contemplate spectrum deconvolution with and without background removal (essentially a straight line nearby the transition under analysis), and data from different measurement runs. According to this approach, in addition to the data dispersion provided by different measurements, the lower (*higher*) end of the error bars correspond to the experimental values achieved with (*without*) background removal.

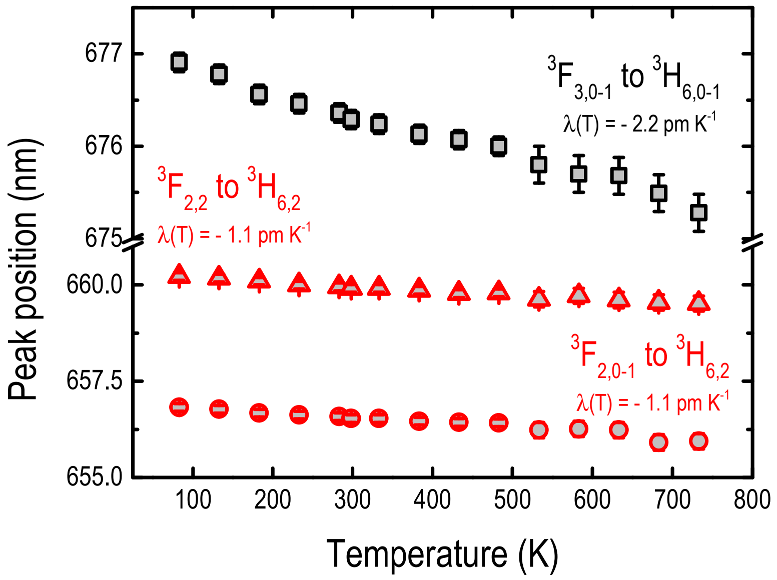

Supplementary Figure S4– Temperature-induced wavelength shift of the visible Tm^{3+}-related transitions as observed from a TiO_2:Tm^{3+} sample. The data were obtained by exciting the samples with 488.0 nm – after 2 min of thermal stabilization. The error bars comprise uncertainties due to experimental acquisition (*i.e.*, spectra resolution and different measurement runs) and data analysis (involving background removal, or not).

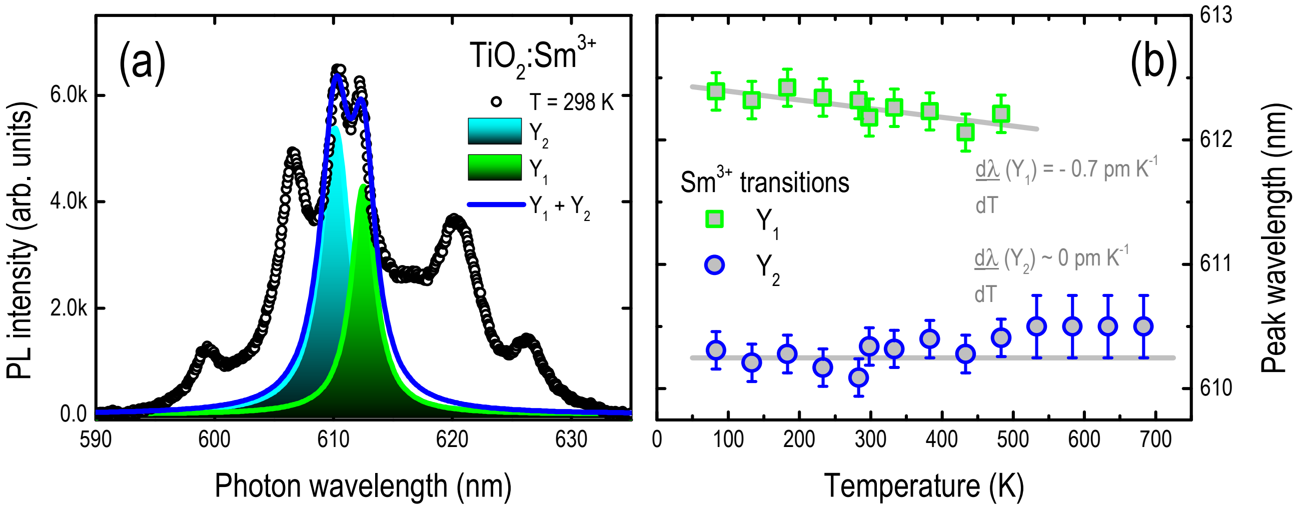

Supplementary Figure S5– (a) Sm^{3+}-related $^4G_{5/2} \rightarrow {}^6H_{7/2}$ transition denoting the Y_1 and Y_2 optical emissions around 610 nm. The spectrum was obtained at room-temperature by exciting the TiO_2:Sm^{3+} sample with 488.0 nm photons. (b) Temperature-induced wavelength shift of the Y_1 and Y_2 emissions, as determined in the 83–673 K range. Above ~ 500 K, background contribution and Y_1-Y_2 lines overlapping prevented a clear analysis of the PL spectra.

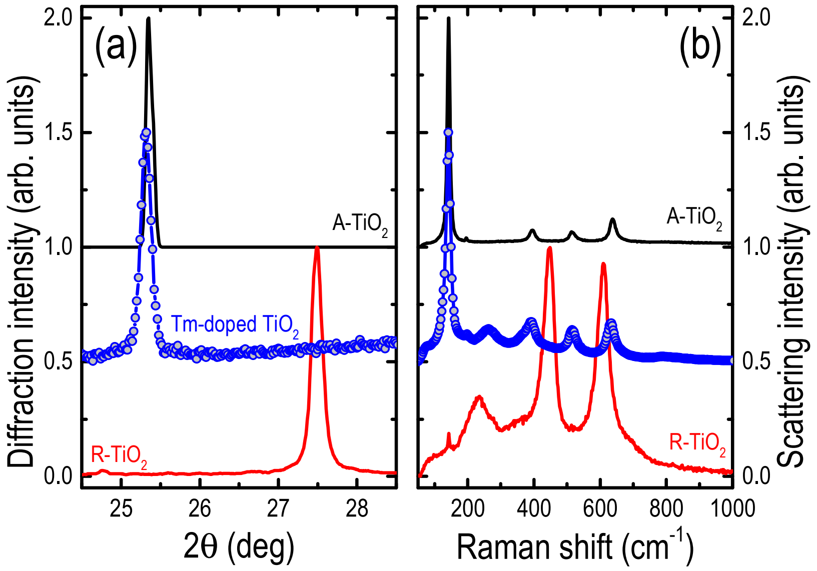

Supplementary Figure S6– (a) X-ray diffractogram of a Tm-doped TiO_2 film, along with the XRD patterns of TiO_2 in the Anatase (A-) and Rutile (R-) phases. (b) Raman scattering spectra of the very same samples presented in (a). The Tm-doped TiO_2 film was thermally annealed for 30 min, under a flow of oxygen gas, at 873 K.
